# Efficacy analysis of self-help position therapy after holmium laser lithotripsy via flexible ureteroscopy

**DOI:** 10.1186/s12894-018-0348-1

**Published:** 2018-05-08

**Authors:** Jie Yang, Rong-zhen Tao, Pei Lu, Meng-xing Chen, Xin-kun Huang, Ke-liang Chen, Ying-heng Huang, Xiao-rong He, Li-di Wan, Jing Wang, Xin Tang, Wei Zhang

**Affiliations:** 1 Department of Urology, First Affiliated Hospital of Nanjing Medical University, Nanjing, 210029 Jiangsu China; 2 First Clinical Medical College, Nanjing Medical University, Nanjing, Jiangsu China; 3 Department of Urology, The Affiliated Jiangning Hospital with Nanjing Medical University, Nanjing, Jiangsu China

**Keywords:** Flexible ureteroscopy, Holmium laser lithotripsy, Self-help, Position therapy, Hydronephrosis

## Abstract

**Background:**

To observe the efficacy of self-help position therapy (SHPT) after holmium laser lithotripsy via flexible ureteroscopy (FURS).

**Methods:**

From January 2010 to November 2015, 736 nephrolithiasis patients who had received FURS lithotripsy were analyzed retrospectively. In position group, 220 cases accepted SHPT after lithotripsies, and 428 cases as control, coming from another independent inpatient area in the same center. The stone-free status (SFS) between two groups were compared at the 2nd, 4th and 12th week ends by X-ray examinations.

**Results:**

The preoperative incidence of hydronephrosis (25.9% vs. 18.0%, *p* = 0.018) or lower calyceal seeper (33.6% vs. 24.3%, *p* = 0.012) and the proportion of patients with > 2.0 cm stones (33.6% vs. 24.3%, *p* = 0.003) were all significantly higher in position group than in control group. There were no substantial difference between two groups in age, BMI, gender and medical histories. In postoperative followup, the incidence of hydronephrosis in position group was significantly lower than in control group (9.5% vs. 15.7%, *p* = 0.032) after removing double-J stents. In position group, the SFS of the 2nd week end (60.9% vs. 47.2%, *p* = 0.001), the 4th week end (74.1% vs. 62.8%, *p* = 0.004) and the 12th week end (86.9% vs. 79.4%, *p* = 0.021) were all significantly higher than those in control group.

**Conclusions:**

SHPT after holmium laser lithotripsy via FURS may increase postoperative SFS, accelerate stone fragment clearance, and decrease the incidence of hydronephrosis after removal of double-J stents. The therapy does not require professional assistance and is economical, simple, and effective.

**Electronic supplementary material:**

The online version of this article (10.1186/s12894-018-0348-1) contains supplementary material, which is available to authorized users.

## Background

Nephrolithiasis is one of the most frequently encountered diseases in urology practice. It varies in occurrence globally [1]. In 2011, the incidence was 1–5% in China and 2–19% in Western countries [2–4]. Minimally invasive procedures are being used more often for the treatment of nephrolithiasis. Flexible ureteroscopy (FURS) with holmium laser lithotripsy is the most popular and mature technology for small- and mid-size renal stones (≤2.5 cm). The documented advantages of FURS include minimal trauma, few complications, and rapid recuperation [5–7]. However, postoperative stone residue and fusion of drainable fragments (≤4 mm) are still intractable problems in clinical practice [8, 9]. Hyams et al. reported 120 cases undergoing FURS with holmium laser lithotripsy for renal stones 2–3 cm in size (mean 2.4 cm). During a 2-month follow-up, 56 (47%) patients were truly stone free, 20 (19%) had a residual burden of < 2 mm, and 24 (17%) had a residual burden of 2–4 mm [10]. Residual lower pole fragments < 2 mm in diameter can still be difficult to clear from the renal collecting system due to gravity and anatomy (the length and width of the lower caliceal infundibulum and the relative angle between the lower calyx and renal pelvis) [11–13].

To address this problem, the inversion-table treatment has been introduced for clinical use [14, 15]. It is a complicated operation requiring professional assistance and is expensive. These drawbacks have hindered its clinical application. The treatment is often unsuitable for patients with severe heart or brain vessel diseases.

Our long-term experience has indicated the value of self-help position therapy (SHPT) to increase the passive egress of stone fragments ≤4 mm in size. SHPT does not require professional assistance and appears to be safe, simple, effective, and suitable for most patients. To investigate its exact efficacy, data of 736 consecutive patients with nephrolithiasis who received holmium laser lithotripsy via FURS from January 2010 to November 2015 were retrospectively analyzed. The patients were divided into a position group and a control group to compare stone-expelling efficacy by postoperative stone free status (SFS) and stone expulsion time.

## Methods

### Patients

**Fig. 1:**
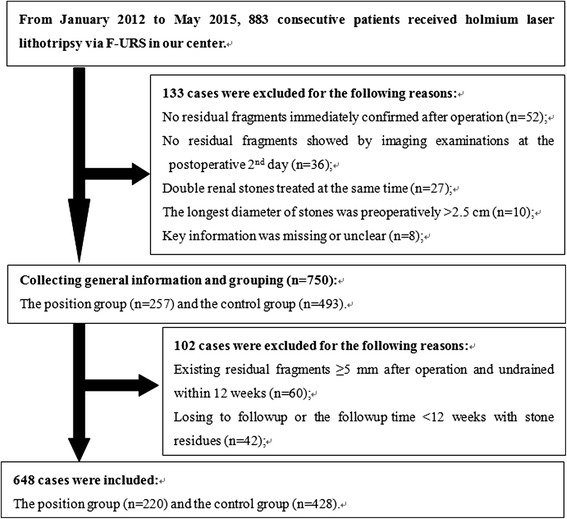
Flowchart for case selection

From January 2010 to November 2015, holmium laser lithotripsies via FURS were performed on a consecutive series of 736 adult patients with kidney stones by the same surgical team in our center. Applying strict inclusion criteria, 648 patients were ultimately included (Fig. 1). Outpatient and inpatient records for each enrollee were reviewed, which included admission and discharge records, laboratory examination reports, radiological images, operative records, and stone analysis reports. Information was collected in telephone or face-to-face interviews using a uniform questionnaire that consisted of general characteristics including population and practices between institutions, personal medical history including lithotripsy and double-J stent placement, lifestyle, occupational history, dietary habits, and family history. Consent for participation was obtained from all participants. This study was approved by the Ethic Committee of First Affiliated Hospital of Nanjing Medical University.

### Perioperative and surgical procedure

All enrollees received a preoperative plain abdominal radiograph of the kidneys, ureters, and bladder (KUB) and unenhanced computed tomography (CT) to assess hydronephrosis and the size, location, and number of stones. The same imaging regimen was used for all patients. Stone size was determined by measuring the longest diameter on the preoperative radiologic images. In the case of multiple stones, the sum of the longest diameters of stones was used [16].

Patients were placed in a lithotomy position after general anesthesia, then a semirigid ureteroscope (8–9.8 F; Richard Wolf GmbH, Knittlingen, Germany) was retrogradely inserted into the upper urinary tract with the assistance of a Zebra guidewire (Cook Medical, Bloomington, IN, USA) to check the ureter and simplify the placement of a 12/14F ureteral access sheath (UAS; Cook Medical, Limerick, Ireland). A 7.5F fiberoptic FURS (P5; Olympus, Tokyo, Japan) was inserted into the renal pelvis via the UAS to search and fragment stones (≤2 mm). Fragmentation was done using a 200 um holmium laser fiber at an energy setting of 12–20 W based on the visually determined hardness of stones. Routinely, a 4.7F double-J stent (Bard Peripheral Vascular, Tempe, AZ, USA) was placed after lithotripsy. If the UAS could not be positioned due to ureter straitness, a 6F double-J stent (Bard Peripheral Vascular) was implanted for 2 weeks before lithotripsy.

**Fig. 2:**
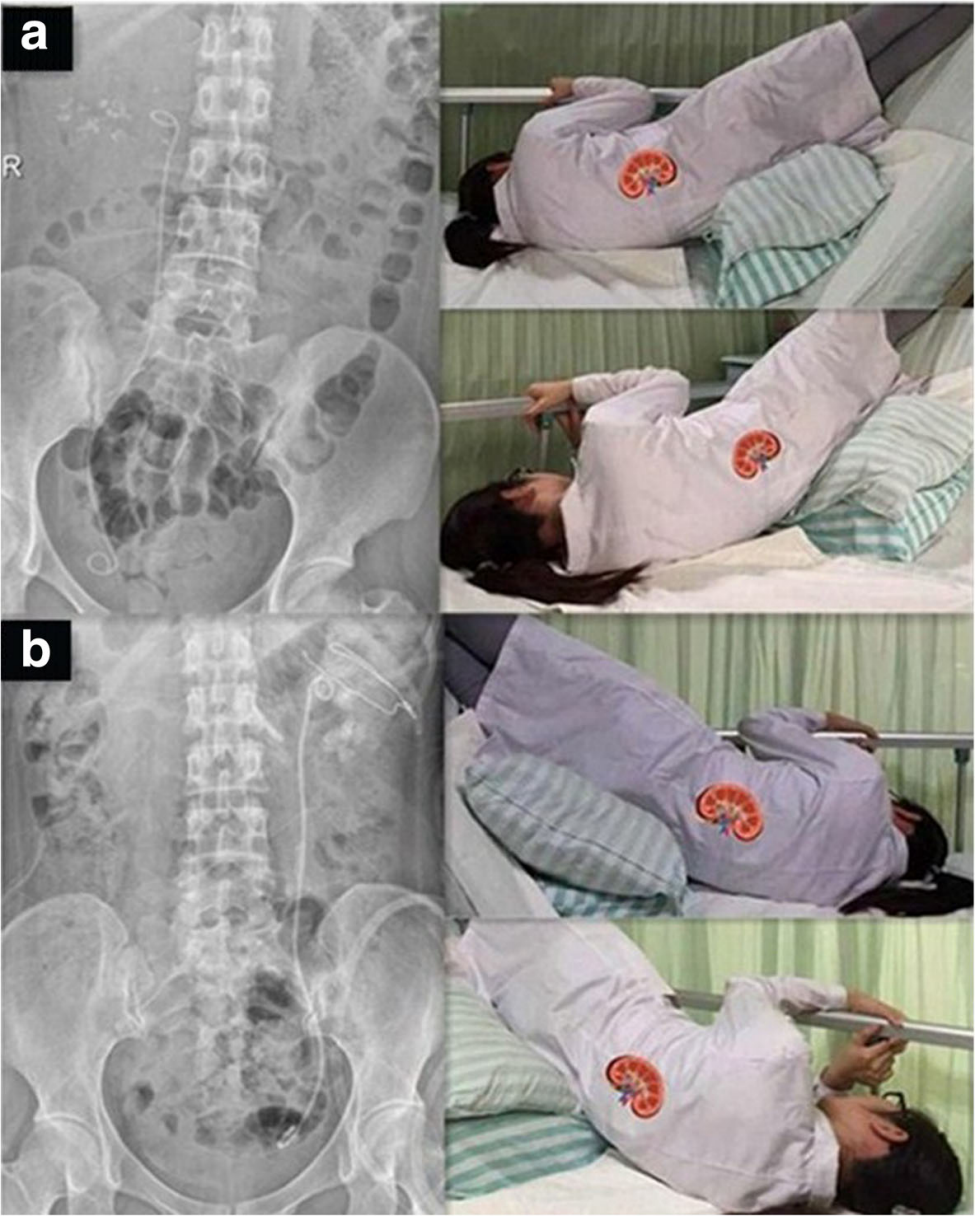
The self-help position therapy (SHPT) after lithotripsies. **a** SHPT for right renal residual fragments; **b** SHPT for left renal residual fragments

A kidney, ureter, and bladder (KUB) examination was done on postoperative day 1. If residual fragments were found, patients in the position group were instructed to assume the SHPT, in which they adopted a contralateral head-down tilt position of at least 45° and maintained the position for 5 min (Fig. 2). The patients were instructed to perform the SHPT once a day during their hospitalization and for 12 weeks following discharge depending on the expelling efficacy of the stone. The control group comprised patients in another independent inpatient area of the same center. They were not given any information regarding position therapy for expelling of fragments. Both groups were suggested to receive medical expulsive therapy consisting of tamsulosin twice a day [17] and hydration with about 2 L of water each day. Implanted double-J stents were removed at postoperative 1–3 months on the basis of the stone-expelling efficacy.

### Follow-up

Follow-up determinations included complete blood count, routine urinalysis, serum creatinine, and KUB examination. The determinations were done at postoperative week 2, 4, and 12 (Additional file 1: Figure S1). SFS was defined as no radiological evidence of stone or the presence of ≤2 mm asymptomatic fragments in the urinary tract [18–20].

### Statistical analyses

SPSS v.16.0 for Windows (IBM Corp., Armonk, NY, USA) was used to perform statistical analyses. Continuous variables are presented as mean ± standard deviation. Patient demographics, follow-up time, and surgical outcomes between the two groups were compared using independent sample t test. The chi-squared test was used to compare other preoperative and postoperative clinical characteristics between the two groups. A *p*-value < 0.05 was considered significant.

## Results

### Demographics and preoperative clinical characteristics

The mean age at diagnosis in the position and control group was 46.4 and 44.1 years, respectively. The incidence of preoperative hydronephrosis (25.9% vs. 18.0%, *p* = 0.018) and lower calyceal seeper (33.6% vs. 24.3%, *p* = 0.012), and the proportion of patients with stones > 2.0 cm in size (33.6% vs. 24.3%, *p* = 0.003) were significantly higher in the position group than in the control group. The percentage of patients with stones that were exclusively located in the lower calyx was slightly and non-significantly higher in the position group than in the control group (47.3% vs. 37.6%, *p* = 0.164). There was no substantial difference between two groups in mean age, body mass index, gender, history of hypertension and diabetes, history of preoperative extracorporeal shock-wave lithotomy (ESWL), and nephrolithiasis surgery, preoperative renal function, and history of double-J stents placement (all *p* > 0.05) (Table 1).

**Table 1:** Comparisons of patients’ demographics and preoperative clinical characteristics between the two groups

Variables, mean ± SD or n (%)	Position group (*n* = 220)	Control group (*n* = 428)	*P* value
Age, year	46.4 ± 5.3	44.1 ± 3.7	0.782
BMI, kg/m2	22.1 ± 2.7	23.3 ± 1.5	0.635
Gender			
Male	123 (55.9)	222 (51.9)	–
Female	97 (44.1)	206 (48.1)	0.329
Hypertension history			
No	160 (72.7)	328 (76.6)	–
Yes	60 (27.3)	100 (23.4)	0.275
Diabetes history			
No	201 (91.4)	383 (89.5)	–
Yes	19 (8.6)	45 (10.5)	0.448
Stone size, cm			
< 1.0 cm	37 (16.8)	106 (24.8)	–
1.0–2.0 cm	109 (49.6)	218 (50.9)	0.108
2.0–2.5 cm	74 (33.6)	104 (24.3)	0.003**
Stone locations			
Pelvis or upper calyx	83 (37.7)	166 (38.8)	–
Middle calyx	33 (15.0)	101 (23.6)	0.077
Lower calyx	104 (47.3)	161 (37.6)	0.164
ESWL history			
No	115 (52.3)	245 (57.2)	–
Yes	105 (47.7)	183 (42.8)	0.228
Nephrolithiasis operation histories^a^			
No	164 (74.5)	297 (69.4)	–
Yes	56 (25.5)	131 (30.7)	0.170
Preoperative hydronephrosis			
No	163 (74.1)	351 (82.0)	–
Yes	57 (25.9)	77 (18.0)	0.018*
Preoperative lower calyceal seeper			
No	146 (66.4)	324 (75.7)	
Yes	74 (33.6)	104 (24.3)	0.012*
Preoperative renal function			
Normal	199 (90.5)	394 (92.0)	–
Abnormal	21 (9.5)	34 (8.0)	0.488
Double-J stents placed histories			
No	140 (63.6)	288 (67.3)	–
Yes	80 (36.4)	140 (32.7)	0.352

*BMI* body mass index, *SD* standard deviation, *ESWL* extracorporeal shock wave lithotripsy

^a^Nephrolithiasis operation histories include flexible ureteroscope lithotripsy, percutaneous nephrolithotomy or nephrolithotomy

*p < 0.05, **p < 0.01

### Surgical outcomes and postoperative clinical characteristics

In postoperative follow-up, the incidence of hydronephrosis in the position group was significantly lower than in the control group (9.5% vs. 15.7%, *p* = 0.032). SFS in the position group was significantly higher than in the control group at week 2 (60.9% vs. 47.2%, *p* = 0.001), week 4 (74.1% vs. 62.8%, *p* = 0.004), and week 12 (86.9% vs. 79.4%, *p* = 0.021). However, there was no statistical significance between the groups concerning operative time, HGB decrease, hospital stay, postoperative anal aerofluxus time, stone composition, and postoperative renal function (all *p* > 0.05) (Table 2).

**Table 2:** Comparisons of surgical outcomes and postoperative clinical characteristics between two groups

Variables, mean ± SD or *n* (%)	Position group (*n* = 220)	Control group (*n* = 428)	*P* value
Follow-up, month	3.3 ± 0.4	3.6 ± 0.4	0.417
Surgical outcomes			
operative time, hour	1.7 ± 0.6	1.5 ± 0.5	0.243
HGB decrease (g/L)	4.9 ± 3.4	5.3 ± 3.7	0.915
Hospital stay, day	1.8 ± 0.4	1.9 ± 0.5	0.973
Postoperative anal aerofluxus time, day	1.2 ± 0.3	1.1 ± 0.4	0.853
Stone compositions			
Calcium oxalate	127 (57.7)	257 (60.0)	–
Struvite	51 (23.2)	84 (19.6)	0.322
Calcium phosphate	42 (19.1)	87 (20.4)	0.914
Postoperative renal function			
Normal	209 (95.1)	410 (95.8)	–
Abnormal	11 (4.9)	18 (4.2)	0.643
Postoperative hydronephrosis			
No	199 (90.5)	361 (84.3)	–
Yes	21 (9.5)	67 (15.7)	0.032*
SFS at the 2nd week end			
No	86 (39.1)	226 (52.8)	–
Yes	134 (60.9)	202 (47.2)	0.001**
SFS at the 4th week end			
No	57 (25.9)	159 (37.1)	
Yes	163 (74.1)	269 (62.9)	0.004**
SFS at the 12th week end			
No	29 (13.1)	88 (20.6)	
Yes	191 (86.9)	340 (79.4)	0.021*

*SD* standard deviation, *HGB* hemoglobin, *SFS* stone free status

*p < 0.05, **p < 0.01

## Discussion

The clinical application of FURS was first reported in 1964 [21]. Since then, the equipment and optical imaging system have evolved quickly. FURS with holmium laser, which allows retrograde access to any calices of the renal collecting system, has increasingly become the first-line therapy for renal calculi, especially for patients with blood coagulation dysfunction, renal insufficiency, obesity, isolated kidney, or undergoing failed lithotripsies of ESWL or percutaneous nephrolithotomy (PCNL) [22–24].

The SFR is 70–90% after holmium laser lithotripsy using FURS at 3 months postoperatively [8, 9]. However, residual stone and gravel fusion remain troublesome problems. Residual calculi ≤2 mm in size in the absence of obstruction or infection are defined as clinically insignificant residual fragments [19, 20]. Although these fragments are usually clinically insignificant, they might enlarge and lead to infection or obstruction of the urinary tract. In a study of 384 patients undergoing FURS, clinically insignificant residual fragments were present in 44 (11.5%) patients by abdominal CT from postoperative 3 weeks to 3 months [25]. Among them, 15 patients showed symptoms resulting from the enlargement or fusion of residual gravels. Therefore, promoting the discharge of clinically insignificant residual fragments from the urinary tract as soon as possible is prudent following lithotripsy.

Methods promoting the elimination of residual gravel and increasing postoperative SFR include drug-mediated dissolution of stones or stone discharge by movement or an inverted position. Position therapy has proven especially effective for lower pole residual gravel. Lower calices are the lowest parts of the renal collecting system, and are usually 2–3 cm distant from the calyceal bottom to the pelvic openings [26]. The distance will increase significantly with seeper in lower calices. Therefore, the lower pole residual stones are commonly difficult to expel with urine in the upright position due to the gravity and postoperative hydronephrosis, even if they have broken into small (< 2 mm) pieces. The effectiveness of inversion positioning prompted our center to initiate SHPT after holmium laser lithotripsy via FURS beginning in 2010 (Fig. 2), particularly for patients with preoperative hydronephrosis (Table 1).

Postoperative position therapy is also imperative for patients with large kidney stones (≤4 mm) after lithotripsy. If the stone pieces are not excreted during the first 3 months postoperatively, they will tend to fuse or growth, which can result in obstruction or infection in the urinary tract. Presently, although the preoperative rates of hydronephrosis and lower calyceal seeper were significantly higher in the position group than in the control group, the incidence of hydronephrosis was conspicuously lower in the position group after removal of double-J stents (Table 2). This highlights the importance of the timely expulsion of residual stones.

The discharge efficacy of residual stones with the assistance of inversion-table treatment has been confirmed. Pace et al. reported that a 60-degree tilt inversion combined with mechanical percussion effectively renders residual lower caliceal region stone-free, with a substantially higher SFR than that of the control group (40% vs. 3%, *p* < 0.01) 3 months after ESWL [14]. Another study also showed that inversion-table treatment group had higher SFR than control group in patients with lower calyceal stones undergoing FURS lithotripsy (97.4% vs. 81.8%, *p* < 0.05) [15]. However, the exact effect of an economic and easy-to-do SHPT has not been described. To provide clarity, the present retrospective study was undertaken to analyze the effect of SHPT in a consecutive cohort at our hospital. The position group still had a significantly higher SFS than the control group, although the preoperative incidence of hydronephrosis or lower calyceal seeper and the proportion of patients with stones > 2.0 cm in size were both higher in position group. Especially, at postoperative week 2, the difference of SFS between the two groups was most significant (60.9% vs. 47.2%, *p* = 0.001). Thereafter the difference gradually lessening, but it remained statistically significant at week 12 (86.9% vs. 79.4%, *p* = 0.021) (Table 2). This indicates that inversion exceeding 45-degrees in a head-down position can speed up the expulsion of residual stones and compensate for the deficiency of gravel deposition in lower calices after renal stones are broken into < 2 mm pieces (the standard of CIRF) using FURS lithotripsy.

Our study chronicles the good curative effect of SHPT after holmium laser lithotripsy via FURS. However, therapeutic planning still needs to be individualized and optimized with respect to start time, frequency, duration, and inversion angles.

The study also has some limitations. It is a retrospective study, lacking the characteristics of random grouping and high homogeneity of patients between groups. Furthermore, we did not compare SHPT with inversion-table treatment in the effectiveness of expelling residual stones. Finally, the study is a single center study with relatively small sample size. There is the possibility of sampling error.

## Conclusions

SHPT after holmium laser lithotripsy via FURS may increase postoperative SFS, accelerate stone fragment clearance, and decrease the incidence of hydronephrosis after removal of double-J stents. The therapy does not require professional assistance and is economical, simple, and effective. A prospective randomized controlled trial should be performed.

## Additional file

Additional file 1: **Figure S1.** Abdominal plain films monitor residual fragment expelling at the postoperative 1st day (A), at the postoperative 2nd week end (B), at the postoperative 4th week end (C), and at the postoperative 12th week end (D). (JPG 151 kb)

## Abbreviations

CIRF: Clinically insignificant residual fragments
CT: Computed tomography
ESWL: Extracorporeal shock-wave lithotomy
FURS: Flexible ureteroscopy
KUB: Kidneys, ureters and bladder
PCNL: Percutaneous nephrolithotomy
SFR: Stone-free rate
SFS: Stone free status
SHPT: Self-help position therapy
