# Supplementary figures and images for: Efficacy analysis of self-help position therapy after holmium laser lithotripsy via flexible ureteroscopy
Source: BMC Urol. 2018 May 8;18:33. doi: 10.1186/s12894-018-0348-1 (PMC5941477; doi:10.1186/s12894-018-0348-1)

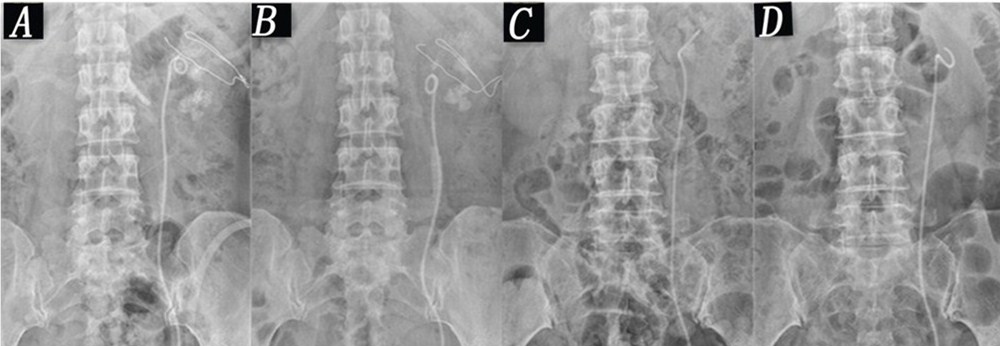

Supplement: Supplementary file 1 — Figure S1. Abdominal plain films monitor residual fragment expelling at the postoperative 1st day (A), at the postoperative 2nd week end (B), at the postoperative 4th week end (C), and at the postoperative 12th week end (D). (JPG 151 kb) [file 12894_2018_348_MOESM1_ESM.jpg]
